# Supplementary material for: MetaCC allows scalable and integrative analyses of both long-read and short-read metagenomic Hi-C data
Source: Nat Commun. 2023 Oct 6;14:6231. doi: 10.1038/s41467-023-41209-6 (PMC10558524; doi:10.1038/s41467-023-41209-6)
Supplement: Supplementary file 1 — Supplementary Information [file 41467_2023_41209_MOESM1_ESM.pdf]

Supplementary Information

MetaCC allows scalable and integrative analyses of both long-read and short-read metagenomic Hi-C data

Yuxuan Du[1] and Fengzhu Sun *[1]

[1]Department of Quantitative and Computational Biology, University of Southern California, USA

Table of contents

Supplementary Notes 2

Supplementary Figures 5

Supplementary Tables 8

Supplementary References 18

*Corresponding author: fsun@usc.com

Supplementary Notes

Supplementary Note 1: The respective performances of HiCzin and HiCBin are markedly deteriorated when only a small fraction of assembled contigs can be annotated

Since both the HiCzin normalization and the HiCBin binning methods require annotating contigs at the species level by TAXAassign to fit their models, we would like to explore how the fraction of annotated contigs affects the respective performances of HiCzin and HiCBin on a synthetic yeast metaHi-C dataset. Details of processing raw data were shown in the Methods section of the main text. Notably, the fraction of annotated contigs from the yeast dataset is much larger than that from other metaHi-C datasets (Supplementary Table 1).

We further downsampled annotated contigs utilized in fitting the HiCzin and HiCBin models to 1% on the yeast dataset, which was not extreme considering that even fewer than 1% of assembled contigs could be labeled on the cow fecal and sheep gut metaHi-C datasets (Supplementary Table 1). We conducted five rounds of downsampling and assessed the respective performances of HiCzin and HiCBin. The performance of HiCzin was measured by the Pearson correlation coefficients between normalized Hi-C contacts and three factors of systematic biases while the results of HiCBin were evaluated using three popular clustering metrics described in Supplementary Note 2. As shown in Supplementary Tables 8 and 9, the respective performances of HiCzin and HiCBin were markedly deteriorated when only one percent of assembled contigs could be annotated.

Supplementary Note 2: Evaluation criteria of the clustering results

The Fowlkes-Mallows score: The Fowlkes-Mallows score (F-score) is defined as the geometric mean of the precision and recall, i.e,

$$\text{FM} = \sqrt{\frac{TP}{TP+FP} \cdot \frac{TP}{TP+FN}}, \tag{S1}$$

where TP is the number of true positives, FP is the number of false positives, and FN is the number of false negatives.

The Adjusted Rand Index: The rand index (RI) is defined as the percentage of correct decisions made by the clustering algorithm, i.e.,

$$\text{RI} = \frac{TP+TN}{TP+TN+FP+FN}, \tag{S2}$$

where TP is the number of true positives, TN is the number of true negatives, FP is the number of false positives, and FN is the number of false negatives.

Then, the Adjusted Rand Index (ARI) can be defined as

$$\text{ARI} = \frac{\text{RI} - \mathbb{E}(\text{RI})}{\max(\text{RI}) - \mathbb{E}(\text{RI})}, \tag{S3}$$

where $\mathbb{E}(\text{RI})$ denotes the Expected Rand Index.

The Normalized Mutual Information: Let U and V denote the sets of true class labels and predicted cluster labels, respectively. Define the entropy of a label set S as

$$H(S) = -\sum_{i=1}^{|S|} P(i)\log(P(i)), \tag{S4}$$

where $P(i) = |S_i|/N$ is the probability of an object in class S_i.

The mutual information (MI) between U and V is calculated by:

$$\mathrm{MI}(U,V) = \sum_{i=1}^{|U|}\sum_{j=1}^{|V|} P(i,j)\log(\frac{P(i,j)}{P(i) \times P'(j)}), \tag{S5}$$

where $P(i,j) = |U_i \cap V_j|/N$, $P(i) = |U_i|/N$, and $P'(j) = |V_j|/N$.

Then, the Normalized Mutual Information (NMI) is defined as

$$\mathrm{NMI}(U,V) = \frac{2 \times \mathrm{MI}(U,V)}{H(U) + H(V)}. \tag{S6}$$

Supplementary Note 3: Polishing HiFi assemblies using short reads did not improve the binning performance on the sheep gut dataset

Pilon [1] polishing of the HiFi assembly from the sheep gut long-read metaHi-C dataset was accomplished using the Illumina short reads derived from the same sheep gut sample with the '–fix indels –nostrays' setting. We then aligned paired-end Hi-C reads to polished contigs as described in the Methods section of the main text and binned contigs using the MetaCC framework. As shown in Supplementary Fig. 2, the polishing step did not substantially improve the binning results.

Supplementary Note 4: The spurious contact removal step with default threshold consistently improved the downstream binning results

To assess the impact of the spurious contact detection step using our default threshold on the subsequent binning process, we executed the MetaCC pipeline without the spurious contact removal step. As a result, the MetaCC binning without spurious contact detection retrieved 75, 101, 6, and 412 near-complete MAGs from the human gut, wastewater, cow rumen, and sheep gut metaHi-C datasets, respectively. With the inclusion of the spurious contact detection step, these numbers were improved to 79, 103, 8, and 417, respectively. Moreover, the total count of high-quality MAGs recovered from the four datasets were also increased from 118, 205, 68, and 696 to 124, 209, 71, and 708, respectively, after the spurious contact removal. These results demonstrated a consistent enhancement of the spurious contact removal step in the downstream binning outcomes for real datasets.

Supplementary Note 5: A standard read cleaning procedure

Adaptor sequences were removed by bbduk from the BBTools suite (v37.25) [2] with parameters 'ktrim=r k=23 mink=11 hdist=1 minlen=50 tpe tbo' and reads were quality-trimmed using bbduk with parameters 'trimq=10 qtrim=r ftm=5 minlen=50'. Then, the first 10 nucleotides of each read were trimmed by bbduk with parameter 'ftl=10'. Identical PCR optical and tile-edge duplicates for Hi-C paired-end reads were removed by the script 'clumpify.sh' from the BBTools suite (v37.25) with default parameters.

Supplementary Note 6: Estimating the number of genomes existing in the metagenomic data using single-copy marker genes

Following the strategy in [3], we utilized single-copy marker genes to estimate the number of genomes in the microbial sample. To predict genes from the contigs, FragGeneScan [4] was employed, and

the predicted genes were scanned using HMMER3 (v3.3.2) [5] with parameter '-cut_tc' to identify 107 single-copy marker genes that are conserved in 95% of sequenced bacteria [6]. After filtering out genes that do not meet the coverage threshold (set at 40%), we determined the number of genomes present in the metagenomic data k as the median number of contigs containing each of the marker genes. This step accounted for the possibility of marker genes being fragmented into multiple pieces, which could affect the estimation of the number of genomes.

Supplementary Note 7: Identifying the species identity of assembled contigs on the synthetic yeast dataset

We first downloaded the reference genomes of all 16 yeast strains from 13 yeast species in the synthetic yeast sample (Supplementary Table 10). As the analyses were made at the species level, the genomes of four strains (FY, CEN.PK, RM11-1A and SK1) from the same species (*Saccharomyces cerevisiae*) were combined into one reference genome. Then, all contigs were aligned to those 13 reference genomes of all known species by BLAST [7] with parameters '-perc identity 95 -evalue 1e-30 -word size 50'. The true species identity of the assembled contigs could be determined if there existed any alignment of the contigs to the species' reference genome (Supplementary Fig. 3).

Supplementary Figures

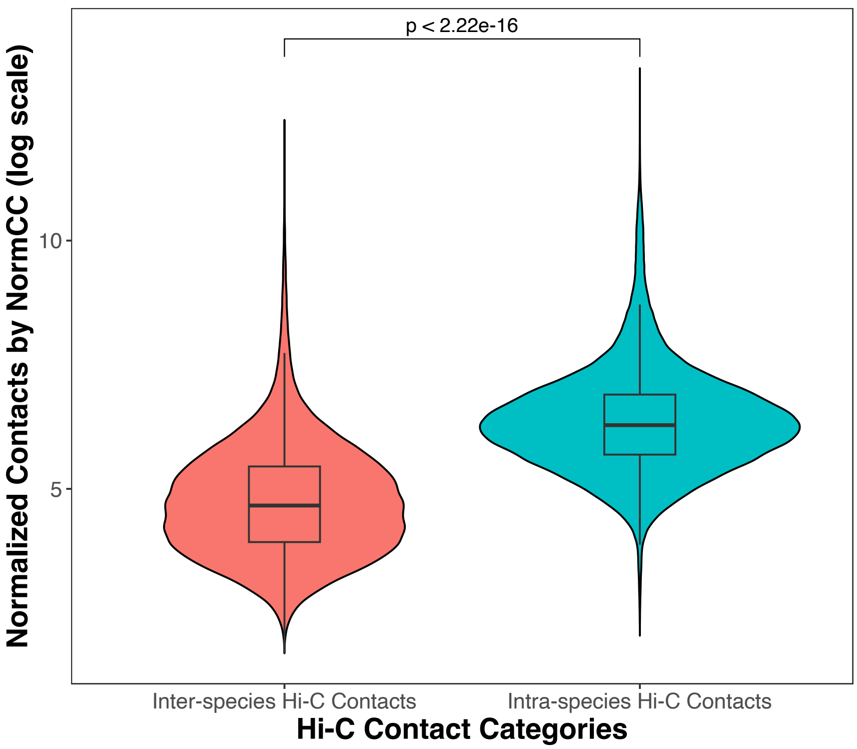

Supplementary Fig. 1 The inter-species versus intra-species Hi-C contacts within the NormCC-normalized Hi-C contact matrix from the synthetic yeast metaHi-C dataset. The y-axis represents the logarithmically scaled values of normalized Hi-C contacts by NormCC. An unpaired t test was conducted to compare the values between 393,228 normalized intra-species Hi-C contacts and 125,860 inter-species Hi-C contacts. The resulting p-value is less than 2.22e-16, indicating that the magnitude of intra-species contacts significantly surpasses that of spurious inter-species contacts.

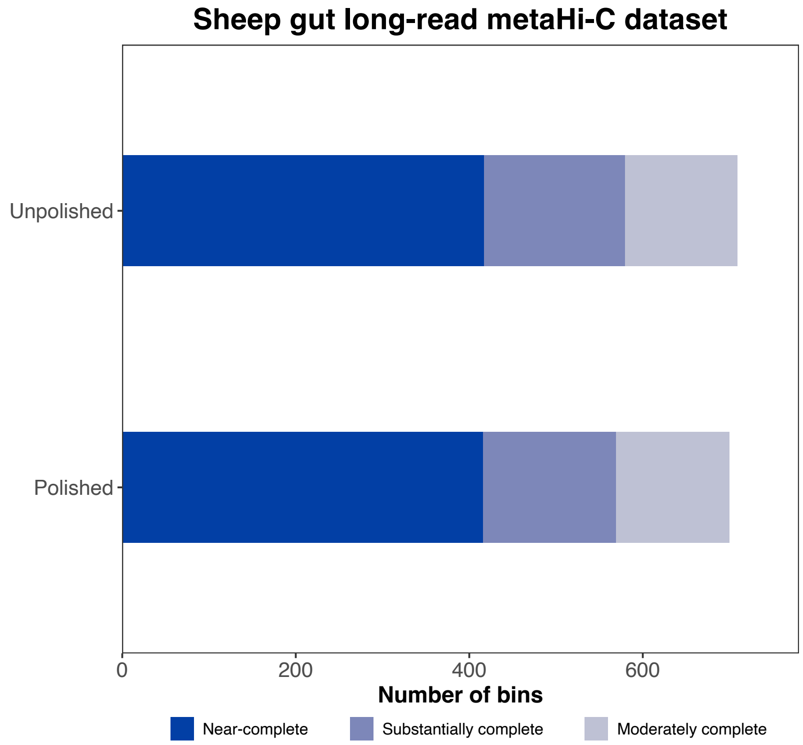

Supplementary Fig. 2 The number of high-quality bins retrieved by MetaCC binning based on unpolished or polished contigs from the sheep gut long-read metaHi-C dataset. Without polishing HiFi assembly using accurate short reads (unpolished), MetaCC binning could retrieve 417, 162, and 130 near-complete, substantially complete, and moderately complete bins, respectively. In contrast, after polishing (polished), only 416, 153, and 131 near-complete, substantially complete, and moderately complete bins were recovered by MetaCC binning, respectively, indicating that the polishing step did not substantially improve the binning results.

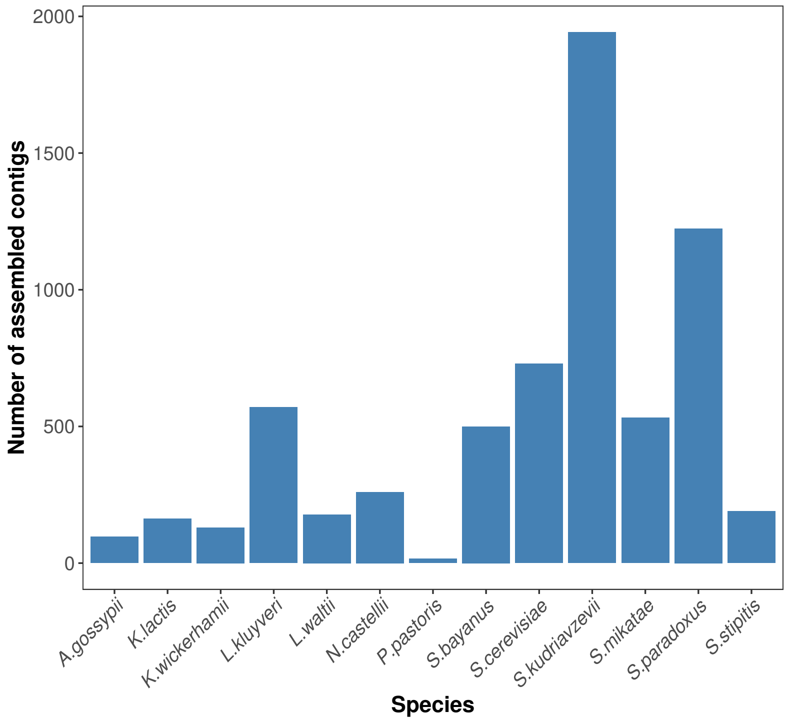

Supplementary Fig. 3 The numbers of assembled contigs from 13 species in the synthetic yeast metaHi-C dataset. The full species names in the x-axis are shown in Supplementary Table 10. Most of the assembled contigs belong to the genus *Saccharomyces*.

Supplementary Tables

Supplementary Table 1. The fractions of annotated contigs for HiCzin and HiCBin on different metaHi-C datasets. Fewer than 1% of assembled contigs could be successfully labeled on the both long-read metaHi-C datasets.

Dataset	The fraction of annotated contigs
Yeast short-read metaHi-C	58.3%
Human gut short-read metaHi-C	11.4%
Wastewater short-read metaHi-C	8.2%
Cow fecal long-read metaHi-C	0.1%
Sheep gut long-read metaHi-C	0.8%

Supplementary Table 2. Comparison of the running time between NormCC and HiCzin on different metaHi-C datasets.

Dataset	HiCzin	NormCC
Yeast	15 min 46 s (6.5 h)	13 s
Human gut	104 min 23 s (7.5 h)	22 s
Wastewater	112 h 31 min (17.7 h)	2 min 30 s
Cow rumen	NA	15 s
Sheep gut	13 s (33.1 h)	4 s

Note: values in the parentheses represent extra time consumed by HiCzin on preparing the input data. NA means that HiCzin failed to converge on the cow rumen metaHi-C dataset.

Supplementary Table 3. Taxonomic statistics of 709 high-quality MAGs retrieved by MetaCC binning from the sheep gut dataset. MAGs were annotated by GTDB-TK at the order level.

The orders of high-quality MAGs	The number of MAGs
Oscillospirales	287
Christensenellales	109
Bacteroidales	89
Lachnospirales	56
RF39	36
TANB77	16
Victivallales	10
Erysipelotrichales	8
Peptostreptococcales	7
Desulfovibrionales	7
RFN20	6
Verrucomicrobiales	5
RF32	5
UBA1381	5
Pirellulales	4
UBA4068	4
Coriobacteriales	3
Monoglobales	3
ML615J-28	3
RFP12	3
Acholeplasmatales	2
Acidaminococcales	2
Burkholderiales	2
Treponematales	2
HGM11327	2
UBA7702	2
UMGS1883	2
Others	15

Supplementary Table 4. The distribution of plasmid contigs among high-quality MAGs retrieved by MetaCC binning from the sheep gut dataset.

The orders of high-quality MAGs	The number of plasmid contigs included
Oscillospirales	39
Bacteroidales	19
Erysipelotrichales	13
RFP12	9
Lachnospirales	4
Christensenellales	3
RF39	3
Peptostreptococcales	2
Enterobacterales	1
Burkholderiales	1
RUG12999	1
DTUO25	1
Coriobacteriales	1
UBA1381	1
HGM11514	1

Supplementary Table 5. The plasmid contigs with coverage $> 2\times$ than the mean average coverage of their respective MAGs retrieved by MetaCC binning from the sheep gut dataset.

Plasmid contig	The coverage of plasmid contigs	The coverage of their respective MAGs
contig_24425	68.94	22.38
contig_61128	158.20	33.93

Supplementary Table 6: The size of shotgun and Hi-C libraries from raw metaHi-C datasets.

Dataset	Size of shotgun libraries (Gbp)	Size of Hi-C libraries (Gbp)
Human gut	37.9	25.9
Wastewater	81.3	28.8
Cow rumen	52	10.1
Sheep gut	255	32.3
Synthetic yeast	17.3	16.2

Supplementary Table 7. The assembly statistics of contigs from all datasets.

Dataset	The number of contigs	Average length (bp)	Total length (bp)
Human gut	105,267	5044	530,969,816
Wastewater	752,580	2539	1,910,562,642
Cow rumen	77,670	13,859	1,076,426,242
Sheep gut	47,246	90,466	4,274,155,803
Synthetic yeast	6,566	19,194	126,030,343

Supplementary Table 8. The normalization performance of HiCzin when only 1% of contigs were annotated on the yeast dataset.

Yeast	Site	Length	Coverage
Without downsampling	0.001	0.001	0.069
Downsampling 1	0.002	0.003	0.207
Downsampling 2	0.001	0.001	0.272
Downsampling 3	0.002	0.002	0.329
Downsampling 4	0.393	0.386	0.006
Downsampling 5	0.004	0.005	0.322

Supplementary Table 9. The binning performance of HiCBin when only 1% of contigs were annotated on the yeast dataset.

Yeast	F-score	ARI	NMI
Without downsampling	0.908	0.894	0.895
Downsampling 1	0.610	0.483	0.697
Downsampling 2	0.761	0.719	0.803
Downsampling 3	0.612	0.485	0.708
Downsampling 4	0.757	0.698	0.848
Downsampling 5	0.850	0.828	0.839

Supplementary Table 10. The species list in the synthetic yeast sample.

Genus	Species	Strain in sample	Reference strain
Saccharomyces	*cerevisiae*	FY4H	FY
Saccharomyces	*cerevisiae*	CEN.PK	CEN.PK
Saccharomyces	*cerevisiae*	RM11-1A	RM11-1A
Saccharomyces	*cerevisiae*	SK1	SK1
Saccharomyces	*paradoxus*	YDG613	YDG613
Saccharomyces	*mikatae*	FM356	IFO 1815
Saccharomyces	*kudriavzevii*	FM527	IFO 1802
Saccharomyces	*bayanus var. uvarum*	YZB5-113	CBS 7001
Naumovozyma	*castellii*	4310	NRRL Y-12630
Lachancea	*waltii*	Kwaltii ura3	NRRL Y-8285
Lachancea	*kluyveri*	FM628	CBS 3082
Kluyveromyces	*lactis*	MW98-8C	NRRL Y-1140
Kluyveromyces	*wickerhamii*	Y-8286	UCD 54-210
Ashbya	*gossypii*	WT	ATCC 10895
Scheffersomyces	*stipitis*	Y-11545	CBS 6054
Pichia	*pastoris*	JC308	GS115

Supplementary References

[1] Bruce J Walker, Thomas Abeel, Terrance Shea, Margaret Priest, Amr Abouelliel, Sharadha Sakthikumar, Christina A Cuomo, Qiandong Zeng, Jennifer Wortman, Sarah K Young, et al. Pilon: an integrated tool for comprehensive microbial variant detection and genome assembly improvement. *PLoS One*, 9(11):e112963, 2014.

[2] Brian Bushnell. BBMap: a fast, accurate, splice-aware aligner. Technical report, Lawrence Berkeley National Lab.(LBNL), Berkeley, CA (United States), 2014.

[3] Yu-Wei Wu, Yung-Hsu Tang, Susannah G Tringe, Blake A Simmons, and Steven W Singer. MaxBin: an automated binning method to recover individual genomes from metagenomes using an expectation-maximization algorithm. *Microbiome*, 2(26), 2014.

[4] Mina Rho, Haixu Tang, and Yuzhen Ye. FragGeneScan: predicting genes in short and error-prone reads. *Nucl Acids Res*, 38(20):e191–e191, 2010.

[5] Robert D Finn, Jody Clements, and Sean R Eddy. HMMER web server: interactive sequence similarity searching. *Nucl Acids Res*, 39(suppl_2):W29–W37, 2011.

[6] Mads Albertsen, Philip Hugenholtz, Adam Skarshewski, Kåre L Nielsen, Gene W Tyson, and Per H Nielsen. Genome sequences of rare, uncultured bacteria obtained by differential coverage binning of multiple metagenomes. *Nat Biotechnol*, 31(6):533–538, 2013.

[7] Mark Johnson, Irena Zaretskaya, Yan Raytselis, Yuri Merezhuk, Scott McGinnis, and Thomas L Madden. NCBI BLAST: a better web interface. *Nucleic Acids Res*, 36(suppl_2):W5–W9, 2008.
