# Supplementary material for: MetaCC allows scalable and integrative analyses of both long-read and short-read metagenomic Hi-C data
Source: Nat Commun. 2023 Oct 6;14:6231. doi: 10.1038/s41467-023-41209-6 (PMC10558524; doi:10.1038/s41467-023-41209-6)
Supplement: Supplementary file 3 — Description of Additional Supplementary Files [file 41467_2023_41209_MOESM3_ESM.pdf]

**File name: Supplementary Data 1**

Description: Detailed results of qc3C for metagenomic Hi-C datasets used in this study.
