# Supplementary material for: MetaCC allows scalable and integrative analyses of both long-read and short-read metagenomic Hi-C data
Source: Nat Commun. 2023 Oct 6;14:6231. doi: 10.1038/s41467-023-41209-6 (PMC10558524; doi:10.1038/s41467-023-41209-6)
Supplement: Supplementary file 5 — Reporting Summary [file 41467_2023_41209_MOESM5_ESM.pdf]

nature portfolio

Corresponding author(s): Fengzhu Sun

Last updated by author(s): Aug 15, 2023

Reporting Summary

Nature Portfolio wishes to improve the reproducibility of the work that we publish. This form provides structure for consistency and transparency in reporting. For further information on Nature Portfolio policies, see our Editorial Policies and the Editorial Policy Checklist.

Statistics

For all statistical analyses, confirm that the following items are present in the figure legend, table legend, main text, or Methods section.

n/a	Confirmed	
☐	☒	The exact sample size (*n*) for each experimental group/condition, given as a discrete number and unit of measurement
☒	☐	A statement on whether measurements were taken from distinct samples or whether the same sample was measured repeatedly
☐	☒	The statistical test(s) used AND whether they are one- or two-sided<br>*Only common tests should be described solely by name; describe more complex techniques in the Methods section.*
☐	☒	A description of all covariates tested
☐	☒	A description of any assumptions or corrections, such as tests of normality and adjustment for multiple comparisons
☐	☒	A full description of the statistical parameters including central tendency (e.g. means) or other basic estimates (e.g. regression coefficient) AND variation (e.g. standard deviation) or associated estimates of uncertainty (e.g. confidence intervals)
☐	☒	For null hypothesis testing, the test statistic (e.g. *F*, *t*, *r*) with confidence intervals, effect sizes, degrees of freedom and *P* value noted<br>*Give P values as exact values whenever suitable.*
☒	☐	For Bayesian analysis, information on the choice of priors and Markov chain Monte Carlo settings
☒	☐	For hierarchical and complex designs, identification of the appropriate level for tests and full reporting of outcomes
☐	☒	Estimates of effect sizes (e.g. Cohen's *d*, Pearson's *r*), indicating how they were calculated

Our web collection on statistics for biologists contains articles on many of the points above.

Software and code

Policy information about availability of computer code

Data collection

SRA-Toolkit (v2.10.8) was used to collect data from the NCBI database.

Data analysis

MetaCC is an open-source pipeline available at https://github.com/dyxstat/MetaCC.

Custom scripts used in this study to process the intermediate data and plot figures are available at https://github.com/dyxstat/Reproduce_MetaCC.

Other software and tools used in this study:
BBTools v37.25
MEGAHIT v1.2.9
Canu v1.6+101 changes (r8513)
metaFlye v2.9
BWA MEM v0.7.17
minimap2 v2.24
SAMtools v1.9
FragGeneScan https://github.com/COL-IU/FragGeneScan.git
HMMER v3.3.2
CheckM v1.1.3
HiCzin v0.1.0
VAMB v3.0.3
bin3C v0.1.1

HiCBin v1.1.0
MetaTOR v1.1.4
qc3C v0.5
GTDB-TK v2.1.0, Release: R207 v2
Mash v2.2
BLAST v2.12.0
Pilon v1.24
MetaBAT2 v2.12.1
PPR-Meta v1.1
Platon v1.6
leidenalg v0.9.0

For manuscripts utilizing custom algorithms or software that are central to the research but not yet described in published literature, software must be made available to editors and reviewers. We strongly encourage code deposition in a community repository (e.g. GitHub). See the Nature Portfolio guidelines for submitting code & software for further information.

Data

Policy information about availability of data

All manuscripts must include a data availability statement. This statement should provide the following information, where applicable:

- Accession codes, unique identifiers, or web links for publicly available datasets
- A description of any restrictions on data availability
- For clinical datasets or third party data, please ensure that the statement adheres to our policy

All the datasets used in this study are publicly available from the NCBI Sequence Read Archive database (http://www.ncbi.nlm.nih.gov/sra). The human gut dataset used in this study is available under accession codes: shotgun library SRR6131123, Hi-C libraries SRR6131122 and SRR6131124. The wastewater dataset is available under accession codes: shotgun library SRR8239393 and Hi-C library SRR8239392. The cow rumen dataset used in this study is available under accession codes: BioProject PRJNA507739. The sheep gut dataset is available under the accession numbers: HiFi reads SRX10647529 and SRX7628648, Hi-C reads SRX10704191, and WGS short reads SRX7649993. The synthetic yeast sample used in this study is available under the accession codes: shotgun library SRR1263009 and Hi-C library SRR1262938. The final assembly from the cow rumen dataset is available at https://figshare.com/articles/usda_pacbio_second_pilon_indelsonly_fa_gz/8323154. The assembly of PacBio HiFi long reads from the sheep gut dataset is available at https://doi.org/10.5281/zenodo.5228989 under the file `flye.v29.sheep_gut.hifi.250g.fasta.gz'. The curated nucleotide reference database of TAXAssign is available at http://userweb.eng.gla.ac.uk/umer.ijaz/bioinformatics/db.sqlite.gz. The curated nucleotide reference database of TAXAassign is available at http://userweb.eng.gla.ac.uk/umer.ijaz/bioinformatics/db.sqlite.gz. The GTDB-TK reference database is available at https://data.gtdb.ecogenomic.org/releases/release207/207.0/auxillary_files/gtdbtk_r207_v2_data.tar.gz. The UHGG catalogs are available from the MGnify FTP site http://ftp.ebi.ac.uk/pub/databases/metagenomics/mgnify_genomes/human-gut/v1.0/uhgg_catalogue. The NCBI RefSeq database is available at https://ftp.ncbi.nlm.nih.gov/refseq/release. The complete sequence of NCBI plasmid reference genome NZ CP080264.1 is available at https://www.ncbi.nlm.nih.gov/nuccore/NZ CP080264.1. The MAGs generated by MetaCC binning from real metagenomes in the benchmarking can be obtained from Zenodo: https://doi.org/10.5281/zenodo.8057996. The remaining data are available within the Article, Supplementary Information, or Source data. There is no restriction on data availability. Source data are provided with this paper.

Research involving human participants, their data, or biological material

Policy information about studies with human participants or human data. See also policy information about sex, gender (identity/presentation), and sexual orientation and race, ethnicity and racism.

Reporting on sex and gender	This research does not involve human research participants. All human gut data are from publicly available datasets.
Reporting on race, ethnicity, or other socially relevant groupings	This research does not involve human research participants. All human gut data are from publicly available datasets.
Population characteristics	This research does not involve human research participants. All human gut data are from publicly available datasets.
Recruitment	This research does not involve human research participants. All human gut data are from publicly available datasets.
Ethics oversight	This research does not involve human research participants. All human gut data are from publicly available datasets.

Note that full information on the approval of the study protocol must also be provided in the manuscript.

Field-specific reporting

Please select the one below that is the best fit for your research. If you are not sure, read the appropriate sections before making your selection.

☒ Life sciences ☐ Behavioural & social sciences ☐ Ecological, evolutionary & environmental sciences

For a reference copy of the document with all sections, see nature.com/documents/nr-reporting-summary-flat.pdf

Life sciences study design

All studies must disclose on these points even when the disclosure is negative.

Sample size	No sample-size calculation was performed. Since all metagenomic Hi-C experiments used in this study were conducted on a single sample, the sample size remains one for each metaHi-C dataset. We used five short-read and long-read metaHi-C datasets from the synthetic yeast, the human gut, the wastewater, the cow rumen, and the sheep gut environments. The diversity of microbial ecosystems utilized in the benchmarking ensures the sufficiency of the analysis.
Data exclusions	No data were excluded from the analyses.
Replication	The downsampling experiments were replicated five times on annotated contigs, which were utilized to fit the HiCzin and HiCBin models on the synthetic yeast short-read metaHi-C dataset. Each replication of the experiment yielded successful outcomes. The MetaCC framework incorporates relatively deterministic models and employs restrained randomization, ensuring the reproducibility of all results in this study.
Randomization	Since each metagenomic Hi-C dataset was derived from one single sample, there was no assignment of samples into experimental groups and there were no covariates across samples in the same dataset. Therefore, randomization was not relevant to sample allocations and covariate controls.
Blinding	Since each metagenomic Hi-C dataset was derived from one single sample, we neither allocated samples into different experimental groups nor split samples into training and testing. Therefore, the blinding was not relevant to our study.

Reporting for specific materials, systems and methods

We require information from authors about some types of materials, experimental systems and methods used in many studies. Here, indicate whether each material, system or method listed is relevant to your study. If you are not sure if a list item applies to your research, read the appropriate section before selecting a response.

Materials & experimental systems

n/a	Involved in the study	
☒	☐	Antibodies
☒	☐	Eukaryotic cell lines
☒	☐	Palaeontology and archaeology
☒	☐	Animals and other organisms
☒	☐	Clinical data
☒	☐	Dual use research of concern
☒	☐	Plants

Methods

n/a	Involved in the study	
☒	☐	ChIP-seq
☒	☐	Flow cytometry
☒	☐	MRI-based neuroimaging
